# Bis(acetato-κ*O*)[1,2-bis­(2-pyridyl­meth­oxy)benzene-κ^4^ *N*,*O*,*O*′,*N*′]copper(II) tetra­hydrate

**DOI:** 10.1107/S1600536810021495

**Published:** 2010-06-16

**Authors:** Shuang Zhang, Yu-Jie Wang, Dong-Sheng Ma, Ying Liu, Jin-Sheng Gao

**Affiliations:** a Engineering Research Center of Pesticides of Heilongjiang Province, Heilongjiang University, Harbin 150080, People’s Republic of China; b College of Chemistry and Materials Science, Heilongjiang University, Harbin 150080, People’s Republic of China

## Abstract

In the title compound, [Cu(CH_3_COO)_2_(C_18_H_16_N_2_O_2_)]·4H_2_O, the Cu^II^ ion is six-coordinated in a Jahn–Teller-distorted octa­hedral geometry environment defined by four O atoms and two N atoms. A chain structure along [100] is built up by inter­molecular O—H⋯O hydrogen bonds involving the uncoordinated water mol­ecules.

## Related literature

For the synthesis and general backround to flexible pyridyl-based ligands, see: Liu *et al.* (2010*a* ▶,*b* ▶). For a related structure, see: Zhang *et al.* (2010 ▶)
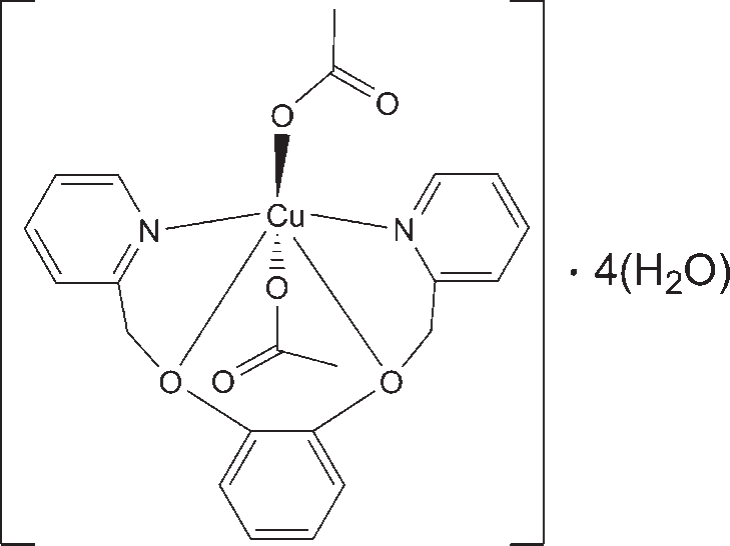


## Experimental

### 

#### Crystal data

[Cu(C_2_H_3_O_2_)_2_(C_18_H_16_N_2_O_2_)]·4H_2_O
*M* *_r_* = 545.02
Triclinic, 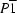

*a* = 8.0192 (16) Å
*b* = 11.291 (2) Å
*c* = 14.117 (3) Å
α = 102.97 (3)°
β = 92.69 (3)°
γ = 93.70 (3)°
*V* = 1240.5 (4) Å^3^
*Z* = 2
Mo *K*α radiation
μ = 0.94 mm^−1^
*T* = 291 K
0.37 × 0.15 × 0.14 mm

#### Data collection

Rigaku R-AXIS RAPID diffractometer
Absorption correction: multi-scan (*ABSCOR*; Higashi, 1995 ▶) *T*_min_ = 0.726, *T*_max_ = 0.880
12216 measured reflections
5621 independent reflections
4677 reflections with *I* > 2σ(*I*)
*R*_int_ = 0.043

#### Refinement

*R*[*F* ^2^ > 2σ(*F* ^2^)] = 0.056
*wR*(*F* ^2^) = 0.169
*S* = 1.05
5621 reflections
318 parameters
H-atom parameters constrained
Δρ_max_ = 1.14 e Å^−3^
Δρ_min_ = −0.44 e Å^−3^

### 

Data collection: *RAPID-AUTO* (Rigaku, 1998 ▶); cell refinement: *RAPID-AUTO*; data reduction: *CrystalClear* (Rigaku/MSC, 2002 ▶); program(s) used to solve structure: *SHELXS97* (Sheldrick, 2008 ▶); program(s) used to refine structure: *SHELXL97* (Sheldrick, 2008 ▶); molecular graphics: *SHELXTL* (Sheldrick, 2008 ▶); software used to prepare material for publication: *SHELXL97*.

## Supplementary Material

Crystal structure: contains datablocks I, global. DOI: 10.1107/S1600536810021495/ng2782sup1.cif

Structure factors: contains datablocks I. DOI: 10.1107/S1600536810021495/ng2782Isup2.hkl

Additional supplementary materials:  crystallographic information; 3D view; checkCIF report

## Figures and Tables

**Table 1:** Hydrogen-bond geometry (Å, °)

*D*—H⋯*A*	*D*—H	H⋯*A*	*D*⋯*A*	*D*—H⋯*A*
O10—H67⋯O6^i^	0.85	2.40	3.072 (6)	137
O10—H66⋯O9	0.85	2.07	2.913 (6)	169
O9—H64⋯O8^ii^	0.85	2.26	2.955 (6)	139
O9—H65⋯O8	0.85	2.09	2.828 (6)	145
O8—H63⋯O7^iii^	0.85	2.06	2.874 (5)	162
O8—H62⋯O7	0.85	1.94	2.784 (5)	176
O7—H61⋯O6^iv^	0.85	1.91	2.755 (4)	177
O7—H60⋯O4	0.85	1.94	2.784 (5)	172

Symmetry codes: (i) 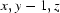; (ii) 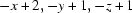; (iii) 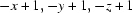; (iv) 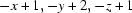.

**Fig. 1.**
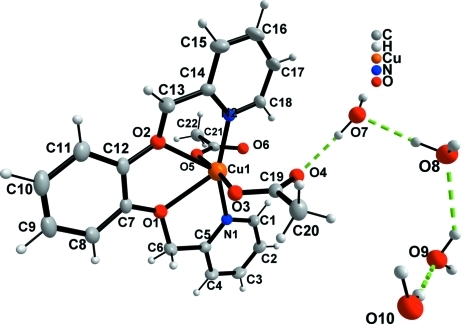
The molecular structure of the title compound, showing displacement ellipsoids at the 30% probability level for non-H atoms.

**Fig. 2.**
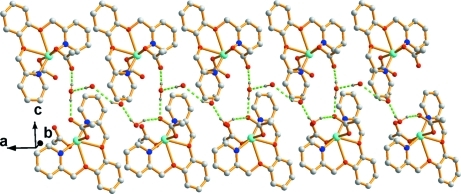
A partial packing view, showing the one-dimensional hydrogen bonding structure along [100] direction. Dashed lines indicate the hydrogen bonds, no involving H atoms have been omitted.

## supplementary crystallographic information

### Comment

N-Heterocyclic ligands coordinated with transition metal ions can form a variety of topology structures, including macrocycles, polyhedra and linear and helical polymers. Our group has report three kinds of flexible pyridyl-based ligands in previous reports (Liu *et al.* 20010*a*; Liu *et al.* 20010*b*). As a part of our continuing work for bipyridyl aromatic ligands, we report the crystal structure of the title compound here, and its analogous monohydrate compound also has been reported by our group (Zhang *et al.* 20010).

1,2-Bis(pyridin-2-ylmethoxy)benzene molecule act as a chelating ligand to coordinate with Cu^II^ ion forming a discrete strucutre. Two acetate counter ions also coordinate to the center Cu^II^ ion, resulting the Cu^II^ ion is six-coordinated in quadrangular bipyramid geometry (Figure 1, Table 1).

A one-dimensional chain structure along [100] direction is built up by intermolecular hydrogen bonds involving the uncoordinated water molecules (Figure 2, Table 2).

### Experimental

The 1,2-Bis(pyridin-2-ylmethoxy)benzene was synthesized by the reaction of ο-dihydroxybenzene and 2-chloromethylpyridine hydrochloride under nitrogen atmosphere and alkaline condition (Liu *et al.*, 2010*a*). Title ligand (0.58 g, 2 mmol) and Cu(CH_3_COO)_2_.H_2_O (0.40 g, 2 mmol) were dissolved in 15 ml e thanol, and then the mixture keep stirring for 30 minute. The resulting solution was filtered, and the filtrate was allowed to stand in a desiccator at room temperature for several days. Bule needle crystals were obtained.

### Refinement

H atoms bound to C atoms were placed in calculated positions and treated as riding on their parent atoms, with C—H = 0.93 Å (aromatic C), C—H = 0.97 Å (methene C), C—H = 0.98 Å (methyl C), and with *U*_iso_(H) = 1.2*U*_eq_(C). Water H atoms were initially located in a difference Fourier map but they were treated as riding on their parent atoms with O—H = 0.85 Å, and with *U*_iso_(H) = 1.5*U*_eq_(O).

### Crystal data

**Table:**

[Cu(C_2_H_3_O_2_)_2_(C_18_H_16_N_2_O_2_)]·4H_2_O	*Z* = 2
*M**_r_* = 545.02	*F*(000) = 568
Triclinic, *P*1	*D*_x_ = 1.459 Mg m^−^^3^
Hall symbol: -P 1	Mo *K*α radiation, λ = 0.71073 Å
*a* = 8.0192 (16) Å	Cell parameters from 10671 reflections
*b* = 11.291 (2) Å	θ = 3.0–27.5°
*c* = 14.117 (3) Å	µ = 0.94 mm^−^^1^
α = 102.97 (3)°	*T* = 291 K
β = 92.69 (3)°	Block, blue
γ = 93.70 (3)°	0.37 × 0.15 × 0.14 mm
*V* = 1240.5 (4) Å^3^	

### Data collection

**Table:**

Rigaku R-AXIS RAPID diffractometer	5621 independent reflections
Radiation source: fine-focus sealed tube	4677 reflections with *I* > 2σ(*I*)
graphite	*R*_int_ = 0.043
ω scan	θ_max_ = 27.5°, θ_min_ = 3.0°
Absorption correction: multi-scan (*ABSCOR*; Higashi, 1995)	*h* = −10→9
*T*_min_ = 0.726, *T*_max_ = 0.880	*k* = −14→14
12216 measured reflections	*l* = −18→18

### Refinement

**Table:**

Refinement on *F*^2^	Primary atom site location: structure-invariant direct methods
Least-squares matrix: full	Secondary atom site location: difference Fourier map
*R*[*F*^2^ > 2σ(*F*^2^)] = 0.056	Hydrogen site location: inferred from neighbouring sites
*wR*(*F*^2^) = 0.169	H-atom parameters constrained
*S* = 1.05	*w* = 1/[σ^2^(*F*_o_^2^) + (0.0738*P*)^2^ + 2.5027*P*] where *P* = (*F*_o_^2^ + 2*F*_c_^2^)/3
5621 reflections	(Δ/σ)_max_ = 0.001
318 parameters	Δρ_max_ = 1.14 e Å^−^^3^
0 restraints	Δρ_min_ = −0.44 e Å^−^^3^

### Special details

**Table:**

Geometry. All e.s.d.'s (except the e.s.d. in the dihedral angle between two l.s. planes) are estimated using the full covariance matrix. The cell e.s.d.'s are taken into account individually in the estimation of e.s.d.'s in distances, angles and torsion angles; correlations between e.s.d.'s in cell parameters are only used when they are defined by crystal symmetry. An approximate (isotropic) treatment of cell e.s.d.'s is used for estimating e.s.d.'s involving l.s. planes.
Refinement. Refinement of *F*^2^ against ALL reflections. The weighted *R*-factor *wR* and goodness of fit *S* are based on *F*^2^, conventional *R*-factors *R* are based on *F*, with *F* set to zero for negative *F*^2^. The threshold expression of *F*^2^ > σ(*F*^2^) is used only for calculating *R*-factors(gt) *etc*. and is not relevant to the choice of reflections for refinement. *R*-factors based on *F*^2^ are statistically about twice as large as those based on *F*, and *R*- factors based on ALL data will be even larger.

### Fractional atomic coordinates and isotropic or equivalent isotropic displacement parameters (Å^2^)

**Table:**

	*x*	*y*	*z*	*U*_iso_*/*U*_eq_	
C1	0.6440 (5)	0.9469 (4)	0.1729 (3)	0.0424 (9)	
H1	0.6547	0.9754	0.2402	0.051*	
C2	0.7766 (5)	0.9716 (4)	0.1190 (4)	0.0486 (10)	
H2	0.8748	1.0144	0.1496	0.058*	
C3	0.7601 (6)	0.9315 (4)	0.0192 (4)	0.0509 (11)	
H3	0.8460	0.9481	−0.0190	0.061*	
C4	0.6143 (5)	0.8665 (4)	−0.0229 (3)	0.0452 (9)	
H4	0.6007	0.8382	−0.0901	0.054*	
C5	0.4872 (5)	0.8432 (3)	0.0357 (3)	0.0340 (7)	
C6	0.3333 (5)	0.7660 (4)	−0.0113 (3)	0.0444 (9)	
H6A	0.3042	0.7845	−0.0736	0.053*	
H6B	0.3543	0.6805	−0.0226	0.053*	
C7	0.0550 (5)	0.7147 (3)	0.0236 (3)	0.0374 (8)	
C8	0.0090 (6)	0.6517 (4)	−0.0710 (3)	0.0475 (10)	
H8	0.0775	0.6576	−0.1213	0.057*	
C9	−0.1418 (6)	0.5795 (4)	−0.0894 (4)	0.0554 (12)	
H9	−0.1733	0.5355	−0.1524	0.066*	
C10	−0.2436 (6)	0.5729 (4)	−0.0153 (4)	0.0566 (12)	
H10	−0.3444	0.5251	−0.0286	0.068*	
C11	−0.1981 (5)	0.6369 (4)	0.0798 (4)	0.0491 (10)	
H11	−0.2684	0.6331	0.1298	0.059*	
C12	−0.0462 (5)	0.7062 (4)	0.0988 (3)	0.0393 (8)	
C13	−0.0439 (6)	0.7318 (5)	0.2704 (3)	0.0531 (11)	
H13	−0.1295	0.6714	0.2681	0.064*	
C14	0.0544 (5)	0.8017 (4)	0.3605 (3)	0.0410 (9)	
C15	−0.0073 (7)	0.8020 (6)	0.4506 (4)	0.0633 (14)	
H15	−0.1091	0.7596	0.4546	0.076*	
C16	0.0828 (8)	0.8653 (7)	0.5345 (4)	0.0775 (18)	
H16	0.0435	0.8652	0.5955	0.093*	
C17	0.2324 (6)	0.9287 (6)	0.5260 (3)	0.0640 (14)	
H17	0.2947	0.9740	0.5809	0.077*	
C18	0.2869 (5)	0.9233 (4)	0.4345 (3)	0.0466 (10)	
H18	0.3892	0.9641	0.4289	0.056*	
C19	0.4783 (5)	0.6757 (4)	0.2747 (3)	0.0357 (8)	
C20	0.5151 (6)	0.5454 (4)	0.2706 (4)	0.0516 (11)	
H20A	0.6264	0.5435	0.2981	0.077*	
H20B	0.5062	0.5003	0.2040	0.077*	
H20C	0.4360	0.5096	0.3070	0.077*	
C21	0.2987 (5)	1.1144 (4)	0.2719 (3)	0.0382 (8)	
C22	0.2136 (7)	1.2277 (4)	0.2681 (4)	0.0553 (11)	
H22A	0.1609	1.2554	0.3280	0.083*	
H22B	0.1304	1.2103	0.2147	0.083*	
H22C	0.2948	1.2899	0.2593	0.083*	
Cu1	0.31166 (5)	0.85895 (4)	0.22267 (3)	0.02996 (15)	
N1	0.5010 (4)	0.8839 (3)	0.1325 (2)	0.0337 (6)	
N2	0.2005 (4)	0.8624 (3)	0.3531 (2)	0.0362 (7)	
O1	0.2007 (3)	0.7897 (3)	0.0502 (2)	0.0424 (6)	
O2	0.0142 (3)	0.7724 (3)	0.1897 (2)	0.0425 (6)	
O3	0.3666 (3)	0.6916 (2)	0.2120 (2)	0.0389 (6)	
O4	0.5540 (4)	0.7602 (3)	0.3366 (2)	0.0463 (7)	
O5	0.2292 (3)	1.0156 (2)	0.2193 (2)	0.0397 (6)	
O6	0.4298 (4)	1.1202 (3)	0.3240 (2)	0.0528 (8)	
O7	0.5369 (4)	0.6904 (3)	0.5132 (2)	0.0563 (8)	
H60	0.5492	0.7168	0.4619	0.084*	
H61	0.5436	0.7494	0.5629	0.084*	
O8	0.7329 (4)	0.4974 (4)	0.5198 (3)	0.0690 (10)	
H62	0.6766	0.5576	0.5164	0.103*	
H63	0.6688	0.4337	0.5166	0.103*	
O9	0.9449 (5)	0.4636 (4)	0.3630 (3)	0.0803 (12)	
H64	1.0478	0.4888	0.3703	0.120*	
H65	0.9111	0.4532	0.4169	0.120*	
O10	0.7313 (7)	0.2533 (5)	0.2556 (3)	0.1047 (17)	
H66	0.8015	0.3131	0.2805	0.157*	
H67	0.6436	0.2585	0.2873	0.157*	

### Atomic displacement parameters (Å^2^)

**Table:**

	*U*^11^	*U*^22^	*U*^33^	*U*^12^	*U*^13^	*U*^23^
C1	0.0347 (19)	0.047 (2)	0.043 (2)	−0.0025 (17)	0.0035 (16)	0.0075 (17)
C2	0.035 (2)	0.044 (2)	0.066 (3)	−0.0034 (18)	0.0084 (19)	0.011 (2)
C3	0.045 (2)	0.052 (3)	0.062 (3)	0.005 (2)	0.023 (2)	0.022 (2)
C4	0.049 (2)	0.049 (2)	0.041 (2)	0.0062 (19)	0.0137 (18)	0.0161 (18)
C5	0.0377 (18)	0.0326 (17)	0.0351 (18)	0.0057 (15)	0.0077 (15)	0.0129 (14)
C6	0.046 (2)	0.052 (2)	0.0335 (19)	−0.0015 (19)	0.0022 (16)	0.0075 (17)
C7	0.0344 (18)	0.0339 (18)	0.042 (2)	0.0034 (15)	−0.0082 (15)	0.0077 (15)
C8	0.047 (2)	0.046 (2)	0.045 (2)	0.0072 (19)	−0.0079 (18)	0.0032 (18)
C9	0.054 (3)	0.044 (2)	0.061 (3)	0.002 (2)	−0.023 (2)	0.002 (2)
C10	0.049 (2)	0.043 (2)	0.074 (3)	−0.011 (2)	−0.024 (2)	0.014 (2)
C11	0.041 (2)	0.048 (2)	0.059 (3)	−0.0042 (19)	−0.0073 (19)	0.019 (2)
C12	0.0365 (19)	0.0387 (19)	0.043 (2)	0.0022 (16)	−0.0097 (16)	0.0133 (16)
C13	0.042 (2)	0.072 (3)	0.045 (2)	−0.017 (2)	0.0033 (18)	0.017 (2)
C14	0.0379 (19)	0.049 (2)	0.040 (2)	0.0039 (17)	0.0098 (16)	0.0166 (17)
C15	0.052 (3)	0.089 (4)	0.053 (3)	−0.008 (3)	0.019 (2)	0.025 (3)
C16	0.071 (3)	0.126 (6)	0.038 (3)	−0.004 (4)	0.015 (2)	0.027 (3)
C17	0.056 (3)	0.106 (4)	0.029 (2)	0.006 (3)	0.0026 (19)	0.013 (2)
C18	0.039 (2)	0.067 (3)	0.035 (2)	−0.001 (2)	−0.0006 (16)	0.0138 (19)
C19	0.0317 (17)	0.0396 (19)	0.0384 (19)	−0.0005 (15)	0.0050 (15)	0.0149 (15)
C20	0.057 (3)	0.043 (2)	0.058 (3)	0.004 (2)	−0.005 (2)	0.0181 (19)
C21	0.045 (2)	0.0383 (19)	0.0320 (18)	0.0038 (17)	0.0093 (16)	0.0088 (15)
C22	0.071 (3)	0.039 (2)	0.056 (3)	0.012 (2)	0.004 (2)	0.0091 (19)
Cu1	0.0289 (2)	0.0340 (2)	0.0271 (2)	−0.00067 (17)	0.00117 (15)	0.00810 (16)
N1	0.0333 (15)	0.0363 (16)	0.0333 (15)	0.0024 (13)	0.0064 (12)	0.0110 (12)
N2	0.0317 (15)	0.0482 (18)	0.0315 (15)	0.0077 (14)	0.0041 (12)	0.0134 (13)
O1	0.0344 (13)	0.0516 (16)	0.0367 (14)	−0.0029 (12)	−0.0009 (11)	0.0033 (12)
O2	0.0395 (14)	0.0478 (16)	0.0391 (15)	−0.0092 (13)	−0.0037 (11)	0.0121 (12)
O3	0.0397 (14)	0.0387 (14)	0.0383 (14)	0.0009 (12)	−0.0009 (11)	0.0102 (11)
O4	0.0382 (14)	0.0456 (16)	0.0517 (17)	−0.0044 (13)	−0.0063 (12)	0.0082 (13)
O5	0.0429 (15)	0.0357 (14)	0.0405 (14)	0.0025 (12)	0.0037 (11)	0.0084 (11)
O6	0.0521 (18)	0.0500 (18)	0.0505 (18)	0.0040 (15)	−0.0066 (14)	0.0011 (14)
O7	0.074 (2)	0.0462 (17)	0.0457 (17)	0.0013 (16)	−0.0037 (15)	0.0061 (13)
O8	0.0480 (19)	0.069 (2)	0.088 (3)	0.0019 (17)	0.0032 (18)	0.014 (2)
O9	0.073 (3)	0.088 (3)	0.075 (3)	0.006 (2)	−0.002 (2)	0.009 (2)
O10	0.126 (4)	0.099 (4)	0.074 (3)	−0.028 (3)	0.023 (3)	−0.006 (3)

### Geometric parameters (Å, °)

**Table:**

C1—N1	1.341 (5)	C16—C17	1.382 (8)
C1—C2	1.386 (6)	C16—H16	0.9300
C1—H1	0.9300	C17—C18	1.373 (6)
C2—C3	1.376 (7)	C17—H17	0.9300
C2—H2	0.9300	C18—N2	1.335 (5)
C3—C4	1.374 (7)	C18—H18	0.9300
C3—H3	0.9300	C19—O4	1.242 (5)
C4—C5	1.390 (5)	C19—O3	1.279 (5)
C4—H4	0.9300	C19—C20	1.508 (6)
C5—N1	1.337 (5)	C20—H20A	0.9600
C5—C6	1.499 (6)	C20—H20B	0.9600
C6—O1	1.406 (5)	C20—H20C	0.9600
C6—H6A	0.9700	C21—O6	1.245 (5)
C6—H6B	0.9700	C21—O5	1.271 (5)
C7—C12	1.382 (6)	C21—C22	1.498 (6)
C7—O1	1.382 (5)	C22—H22A	0.9600
C7—C8	1.385 (6)	C22—H22B	0.9600
C8—C9	1.394 (6)	C22—H22C	0.9600
C8—H8	0.9300	Cu1—O5	1.937 (3)
C9—C10	1.369 (8)	Cu1—O3	1.942 (3)
C9—H9	0.9300	Cu1—N1	2.072 (3)
C10—C11	1.394 (7)	Cu1—N2	2.076 (3)
C10—H10	0.9300	Cu1—O1	2.486 (3)
C11—C12	1.385 (6)	Cu1—O2	2.501 (3)
C11—H11	0.9300	O7—H60	0.8495
C12—O2	1.381 (5)	O7—H61	0.8496
C13—O2	1.408 (5)	O8—H62	0.8487
C13—C14	1.495 (6)	O8—H63	0.8477
C13—H13	0.9300	O9—H64	0.8487
C14—N2	1.339 (5)	O9—H65	0.8493
C14—C15	1.386 (6)	O10—H66	0.8500
C15—C16	1.382 (8)	O10—H67	0.8500
C15—H15	0.9300		
			
N1—C1—C2	123.0 (4)	N2—C18—C17	123.4 (4)
N1—C1—H1	118.5	N2—C18—H18	118.3
C2—C1—H1	118.5	C17—C18—H18	118.3
C3—C2—C1	118.7 (4)	O4—C19—O3	123.7 (4)
C3—C2—H2	120.7	O4—C19—C20	120.3 (4)
C1—C2—H2	120.7	O3—C19—C20	116.0 (3)
C4—C3—C2	118.8 (4)	C19—C20—H20A	109.5
C4—C3—H3	120.6	C19—C20—H20B	109.5
C2—C3—H3	120.6	H20A—C20—H20B	109.5
C3—C4—C5	119.6 (4)	C19—C20—H20C	109.5
C3—C4—H4	120.2	H20A—C20—H20C	109.5
C5—C4—H4	120.2	H20B—C20—H20C	109.5
N1—C5—C4	122.0 (4)	O6—C21—O5	123.6 (4)
N1—C5—C6	119.3 (3)	O6—C21—C22	120.3 (4)
C4—C5—C6	118.7 (4)	O5—C21—C22	116.1 (4)
O1—C6—C5	109.2 (3)	C21—C22—H22A	109.5
O1—C6—H6A	109.8	C21—C22—H22B	109.5
C5—C6—H6A	109.8	H22A—C22—H22B	109.5
O1—C6—H6B	109.8	C21—C22—H22C	109.5
C5—C6—H6B	109.8	H22A—C22—H22C	109.5
H6A—C6—H6B	108.3	H22B—C22—H22C	109.5
C12—C7—O1	115.3 (3)	O5—Cu1—O3	171.42 (11)
C12—C7—C8	120.8 (4)	O5—Cu1—N1	91.93 (12)
O1—C7—C8	123.9 (4)	O3—Cu1—N1	89.79 (12)
C7—C8—C9	118.9 (5)	O5—Cu1—N2	90.02 (13)
C7—C8—H8	120.5	O3—Cu1—N2	91.66 (13)
C9—C8—H8	120.5	N1—Cu1—N2	157.12 (13)
C10—C9—C8	120.3 (4)	O5—Cu1—O1	86.78 (11)
C10—C9—H9	119.8	O3—Cu1—O1	85.82 (11)
C8—C9—H9	119.8	N1—Cu1—O1	71.06 (11)
C9—C10—C11	120.9 (4)	N2—Cu1—O1	131.82 (11)
C9—C10—H10	119.6	O5—Cu1—O2	86.96 (11)
C11—C10—H10	119.6	O3—Cu1—O2	85.71 (11)
C12—C11—C10	118.9 (5)	N1—Cu1—O2	132.86 (11)
C12—C11—H11	120.6	N2—Cu1—O2	70.01 (11)
C10—C11—H11	120.6	O1—Cu1—O2	61.82 (10)
O2—C12—C7	115.2 (3)	C5—N1—C1	118.0 (3)
O2—C12—C11	124.6 (4)	C5—N1—Cu1	123.7 (2)
C7—C12—C11	120.2 (4)	C1—N1—Cu1	118.3 (3)
O2—C13—C14	108.9 (3)	C18—N2—C14	118.7 (3)
O2—C13—H13	125.6	C18—N2—Cu1	117.0 (3)
C14—C13—H13	125.6	C14—N2—Cu1	124.2 (3)
N2—C14—C15	121.0 (4)	C7—O1—C6	116.5 (3)
N2—C14—C13	119.8 (3)	C7—O1—Cu1	122.2 (2)
C15—C14—C13	119.2 (4)	C6—O1—Cu1	110.1 (2)
C16—C15—C14	119.9 (5)	C12—O2—C13	116.7 (3)
C16—C15—H15	120.0	C12—O2—Cu1	121.9 (2)
C14—C15—H15	120.0	C13—O2—Cu1	110.8 (2)
C15—C16—C17	118.6 (5)	C19—O3—Cu1	115.5 (2)
C15—C16—H16	120.7	C21—O5—Cu1	121.8 (3)
C17—C16—H16	120.7	H60—O7—H61	110.2
C18—C17—C16	118.4 (5)	H62—O8—H63	110.9
C18—C17—H17	120.8	H64—O9—H65	109.5
C16—C17—H17	120.8	H66—O10—H67	109.7

### Hydrogen-bond geometry (Å, °)

**Table:**

*D*—H···*A*	*D*—H	H···*A*	*D*···*A*	*D*—H···*A*
O10—H67···O6^i^	0.85	2.40	3.072 (6)	137
O10—H66···O9	0.85	2.07	2.913 (6)	169
O9—H64···O8^ii^	0.85	2.26	2.955 (6)	139
O9—H65···O8	0.85	2.09	2.828 (6)	145
O8—H63···O7^iii^	0.85	2.06	2.874 (5)	162
O8—H62···O7	0.85	1.94	2.784 (5)	176
O7—H61···O6^iv^	0.85	1.91	2.755 (4)	177
O7—H60···O4	0.85	1.94	2.784 (5)	172

Symmetry codes: (i) *x*, *y*−1, *z*; (ii) −*x*+2, −*y*+1, −*z*+1; (iii) −*x*+1, −*y*+1, −*z*+1; (iv) −*x*+1, −*y*+2, −*z*+1.
